# Communities of Fungi in Black Cherry Stumps and Effects of Herbicide

**DOI:** 10.3390/plants9091126

**Published:** 2020-08-31

**Authors:** Robert Korzeniewicz, Marlena Baranowska, Hanna Kwaśna, Gniewko Niedbała, Jolanta Behnke-Borowczyk

**Affiliations:** 1 Department of Silviculture, Poznań University of Life Sciences, Wojska Polskiego 71a, 60-625 Poznań, Poland; korzon@up.poznan.pl (R.K.); marlena.baranowska-wasilewska@up.poznan.pl (M.B.); 2 Department of Forest Pathology, Poznań University of Life Sciences, Wojska Polskiego 71c, 60-625 Poznań, Poland; kwasna@up.poznan.pl (H.K.); jolanta.behnke-borowczyk@up.poznan.pl (J.B.-B.); 3 Institute of Biosystems Engineering, Faculty of Agronomy and Bioengineering, Poznań University of Life Sciences, Wojska Polskiego 50, 60-627 Poznań, Poland

**Keywords:** invasive species, glyphosate, Glifocyd 360 SL, stumps, chemical control, biodiversity of Fungi

## Abstract

So far, there have been no studies on fungal communities in *Prunus serotina* (black cherry) wood. Our objectives were to characterize fungal communities from *P. serotina* wood and to evaluate effects of glyphosate (Glifocyd 360 SL) used on *P. serotina* stumps on abundance, species richness and diversity of those communities. In August 2016, in the Podanin Forest District, stumps of black cherry trees left after felling were treated with the herbicide. Control stumps were treated with water. Wood discs were cut from the surface of the stumps in May and July–August 2017. Eight treatment combinations (2 herbicide treatments × 2 disc sizes × 2 sample times) were tested. Sub-samples were pooled and ground in an acryogenic mill. Environmental DNA was extracted with a Plant Genomic DNA Purification Kit. The ITS1, 5.8S rDNA region was used to identify fungal species, using primers ITS1FI2 5′-GAACCWGCGGARGGATCA-3′ and 5.8S 5′-CGCTGCGTT CTTCATCG-3′. The amplicons were sequenced using the Illumina system. The results were subjected to bioinformatic analysis. Sequences were compared with reference sequences from the NCBI database using the BLASTn 2.8.0 algorithm. Abundance of fungi was defined as the number of Operational Taxonomic Units (OTUs), and diversity as the number of species in a sample. Differences between the number of OTUs and taxa were analyzed using the chi-squared test (χ^2^). Diversity in microbial communities was compared using diversity indices. A total of 54,644 OTUs were obtained. Culturable fungi produced 49,808 OTUs (91.15%), fungi not known from culture had 2571 OTUs (4.70%), non-fungal organisms had 1333 (2.44%) and organisms with no reference sequence in NCBI, 934 OTUs (1.71%). The total number of taxa ranged from 120 to 319. Fungi in stump wood were significantly more abundant in July–August than in May, in stumps >5 cm diameter than in stumps <5 cm diameter, in glyphosate-treated than in untreated stumps when sampled in May, and in untreated than in glyphosate-treated stumps when sampled in July–August. Species richness was significantly greater in July–August than in May, and in stumps >5 cm diameter than in stumps <5 cm diameter, either treated or untreated, depending on size. Herbicides can therefore affect the abundance and diversity of fungal communities in deciduous tree wood. The greater frequency of Ascomycota in herbicide-treated than in untreated stumps indicates their greater tolerance of glyphosate.

## 1. Introduction

Black cherry (*Prunus serotina* Ehrh.) is one of the most pernicious invasive species in forest ecosystems in Europe. It originates from North America and appeared in Europe in the early 17th century. Initially it was an ornamental plant. In the Old World it started to spread on a massive scale after its introduction to European forests in the 19–20th centuries. It is currently present over almost the entire territory of Poland. It spreads mostly in monocultures of Scots pine (*Pinus sylvestris* L.), and plantations of black pine (*Pinus nigra* Arn.) and European larch (*Larix decidua* Mill.) [1,2,3,4,5,6]. Its expansion results from: (i) The popularity of black cherry as an admixture species; (ii) planned and extensive introduction to forests in the 20th century; (iii) high vigor, with rapid growth and development, intensive production of sprouts, abundant production and high viability of seeds and high seed germinability; (iv) vast spatial dispersal of seeds by endozoochory (via ingestion by vertebrate animals, mostly birds and mammals); (v) low habitat requirements and high tolerance of climatic conditions; (vi) absence of natural enemies, including pathogens, that would reduce viability and reproduction outside its natural range [7]. Black cherry can grow in soils that are extremely poor in minerals or in strongly anthropogenically modified habitats [8]. Mass-scale distribution of black cherry in the understorey limits growth and regeneration of native tree species. It also causes degeneration and degradation of the natural habitat [1,2,9].

The conservation of native species by reduction of the population of invasive species is required and promoted worldwide [10]. Elimination of black cherry is a prerequisite for protection of biodiversity and the natural character of Polish forests. Unfortunately, control methods are labor- and time-consuming and expensive. Current strategies for control are usually based on those already found to be most effective. The most common methods include: (i) Mechanical removal of black cherry by felling, pulling, uprooting and girdling, i.e., ring-barking (complete removal of a strip of bark from around the entire circumference of the trunk, which destroys phloem and cambium, responsible for the distribution of sugars and nutrients manufactured in the shoot and for the secondary growth of roots and stem); (ii) chemical removal of plants by herbicide spraying. The two techniques, mechanical and chemical removal, can be applied together. Simultaneous application proved to be effective in the Roztocze and Kampinos National Parks [11,12]. Effectiveness of chemical control with herbicide has been shown previously [13]. Regeneration and re-naturalization of tree species and the herbaceous vegetation layer after application of herbicides has also been observed [3]. Herbicides are also used to control sprouting from felled tree stumps [14].

Although positive effects can result from using herbicides, there are some that may not be used in Poland [12,13]. Substances with mutagenic, carcinogenic, toxic and endocrine-disrupting effects are banned in Polish forests [15].

Glyphosate is effective in killing a wide variety of plants, including grasses and broad-leaved and woody plants. It is an organophosphorus compound, specifically a phosphonate, which acts by inhibiting the plant enzyme 5-enolpyruvylshikimate-3-phosphate synthase. Glyphosate-based herbicide formulations have been used in Europe since the 1970s [16].

Initial reports that glyphosate was harmless to humans and biodegraded in the environment have now been disputed [17]. Herbicides containing glyphosate are widely used in agriculture, horticulture, silviculture, however, surprisingly little is known on potential side effects on non-target organisms, including fungi colonizing wood. Glyphosate reduces the occurrence of mycorrhizal fungi [18] and entomopathogenic fungi [19], but Glyphosate (Roundup), when applied to the soil, usually did not exert any significant effect on the total count of soil fungi [20]. Fungicidal properties of glyphosate formulations varied among fungal species [19].

Efforts towards reducing reliance on herbicides and other methods for reasons of environment, health, economy and sustainability have led to increasing interest in biological approaches to controlling these trees. Fungi can act as biological control agents. For example, *Chondrostereum purpureum* (Pers.) Pouzar (Fungi, Basidiomycota, Agaricales) has a number of characteristics that made it a good candidate as a biological control agent [13,21,22]. It has a broad host range and can be selectively applied through wounding treatments without endangering neighboring trees. The high resistance of conifers and some hardwoods to *C. purpureum* reduces the risk of non-target effects in forests [23]. It occurs naturally within Polish forests and has responded successfully to laboratory methods of culturing [24]. This fungus was used as a mycoherbicide, for example under the brand name BioChon, in order to prevent stump sprouting in hardwoods. It acted as a wood decay promoter. Application of BioChon to control stump sprouting in *P. serotina* generally results in the death of about 95% of trees two years after treatment, and it appears to be effective on aspens, beech, birches, maples, pin cherry and poplars [21].

However, the application of mycoherbicide based on *C. purpureum* is associated with the risk of colonization of nearby fruit or other trees with the fungus, on which it causes silver leaf disease. It is a pathogen of various deciduous trees including species of *Acer*, *Aesculus*, *Alnus*, *Betula*, *Crataegus*, *Fagus*, *Larix*, *Malus*, *Ostrya*, *Populus*, *Prunus*, *Salix*, and *Sorbus.* It attacks mostly species of the rose family (Rosaceae), particularly the genus *Prunus*. Occasionally it also infects conifers (fir, spruce, *Thuja*). Geographically, it is as widespread as its hosts. It is common in woods, orchards and tree plantations in temperate climates. The disease is progressive and often fatal [25,26]. While there is always a level of risk associated with the deployment of a *C. purpureum* as a biological agent, results from previous studies suggest that the risk is small compared with that posed by natural inoculum, and can be minimized by avoiding *C. purpureum*-based treatments in the vicinity of apple orchards [21,23].

Thus, other fungal species are being sought as potential components of mycoherbicides, which could be used in *P. serotina* control [12,27]. Research is being conducted on basidiomycetous, fungi that specialize in the destruction of wood and contribute to elimination of wood from forests [28].

Black cherry wood is colonized by vast communities of fungi which possibly include taxa specializing in the decomposition of wood. Most fungal wood decomposers belong to the Basidiomycota. Most are involved in degradation of the main components of wood: Cellulose, hemicellulose, and lignin. So far, there have been studies on fungal communities in *P. serotina* wood [27,29] or on how they might be affected by chemical treatments. The first such mycological analysis was presented by Marciszewska et al. [27]. Despite this, there are no reports of microfungal communities inhabiting the wood of the black cherry.

The objectives of this study were: (i) To characterize fungal communities from *P. serotina* wood; (ii) to determine the effects of a glyphosate formulation used on *P. serotina* stumps on the abundance, species richness and diversity of fungal communities in the stump wood.

## 2. Results

A total of 54,644 Operational Taxonomic Units (OTUs) were obtained from all wood samples collected from each of the 2-year-old stumps of *P. serotina*. There were 49,808 OTUs (91.15%) of fungi known from cultures, 2571 OTUs (4.70%) of non-cultured fungi, 1333 (2.44%) OTUs of non-fungal organisms, and 934 OTUs (1.71%) of organisms with no reference sequence in the NCBI database. The total number of taxa ranged from 120 to 319. The number of taxa from individual stumps ranged from 83 to 281 (Table 1 and Table S1).

Abundance of fungi in 2-year-old *P. serotina* stump wood measured as number of OTUs was significantly greater in: (i) July–August than in May; (ii) stumps > 5 cm diameter than in stumps < 5 cm diameter; (iii) glyphosate-treated and sampled in May than in untreated, and (iv) untreated and sampled in July–August than in glyphosate-treated (Table 1).

Species richness measured as number of taxa was significantly greater in: (i) July–August than in May; (ii) stumps > 5 cm diameter than in stumps < 5 cm diameter; (iii) glyphosate-treated or untreated depending on wood-disc size (Table 1).

**Figure 1:**
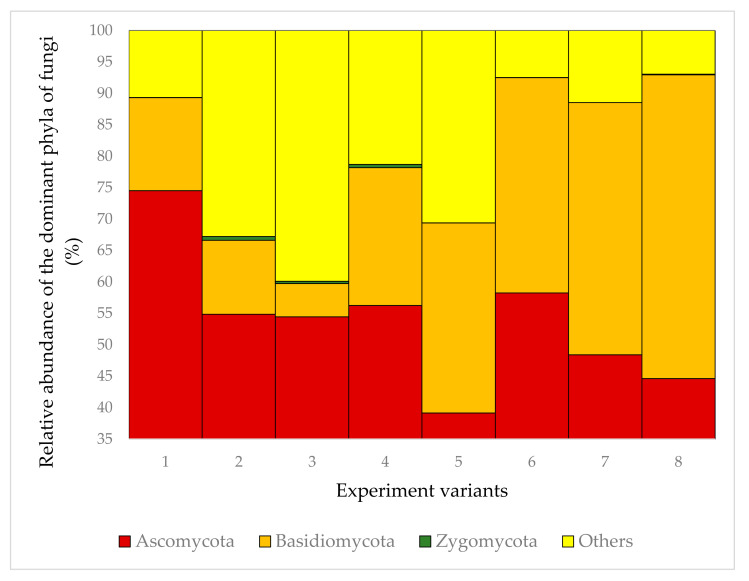
Relative abundance of the dominant phyla of fungi in the 8 experiment variants.

**Figure 2:**
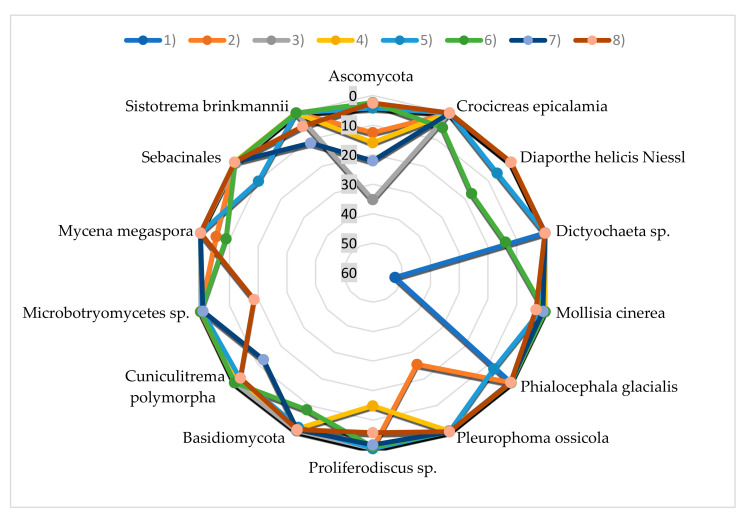
Relative abundance of the dominant taxa of fungi (share above 5%) in the 8 experiment variants.

**Figure 3:**
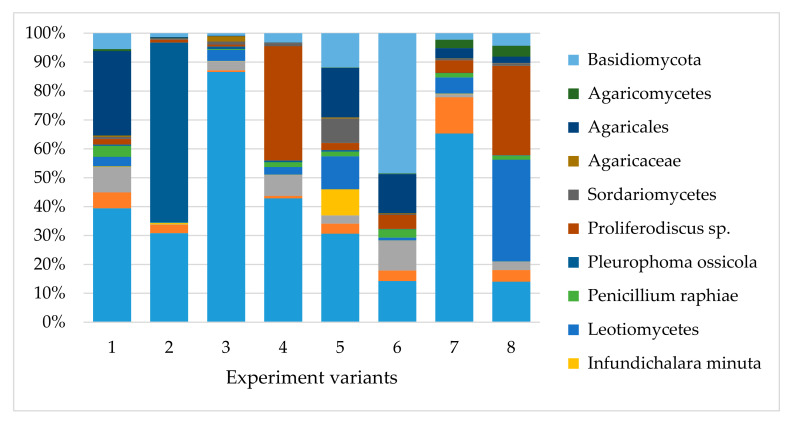
Relative abundance of taxa of the common fungi to all the variants of experience.

Most fungi belonged to phylum Ascomycota (39.15–74.51%; Figure 1), which was represented by 38–128 taxa. The most frequent were: (i) Taxa known to be present in wood, e.g., *Barbatosphaeria* sp., *Cadophora* spp., *Candida* spp., *Cladophialophora* sp., *Coniochaeta* sp., *Crocicreas* spp., *Diaporthe* sp., *Dictyochaeta* sp., *Exophiala* sp., *Jattaea ribicola*, *Phialocephala* sp., *Plectania melastoma*, *Proliferodiscus* sp., *Rhizoscyphus* sp.; (ii) epiphytes known from leaves and bark of trees, e.g., *Infundichalara minuta*, *Mariannaea elegans*, *Sarocladium strictum*, *Yarrowia lipolytica*; (iii) plant pathogens, e.g., *Phacidium grevilleae*; (iv) human pathogens, e.g., *Sarocladium strictum* (Figure 2 and Figure 3).

Basidiomycetes were less abundant (5.30–48.32%; Figure 1) and represented by 18–87 taxa. The most frequent were: (i) Taxa known to be present in wood, e.g., *Auriculariales*, *Ceratobasidium* spp., *Colacogloea* spp., *Cryptococcus* spp., *Curvibasidium cygneicollum*, *Fellozyma inositophila*, *Laetiporus* spp., *Mycena* spp., *Resinicium bicolor*, *Schizophyllum* sp., *Sistotrema brinkmannii*; (ii) plant pathogens, e.g., *Microbotryomycetes* sp., *Trichosporon* spp.; (iii) taxa associated with insects living in wood e.g., *Cuniculitrema polymorpha*, *Hamamotoa lignophila*, *Microbotryomycetes* sp.; (iv) plant pathogens and epiphytes not associated with wood (Figure 2 and Figure 3).

Mean indices for diversity (*D*_Mg_ and *H*′) and evenness (*D* and *E*) of fungal communities were usually less in: (i) May than in July–August (*D*_Mg_ = 27.77 and 32.28, *H*′ = 2.80 and 3.36, *D* = 0.51 and 0.58, *E* = 0.50 and 0.59); (ii) stumps < 5 cm diameter than in stumps > 5 cm diameter (*D*_Mg_ = 27.88 and 32.18, *H*′ = 2.95 and 3.20, *D* = 0.53 and 0.54, *E* = 0.14 and 1.10); (iii) untreated stumps than in glyphosate-treated stumps, determined by two indices (*D*_Mg_ = 28.25 and 31.81, E = 0.08 and 0.15); (iv) glyphosate-treated stumps than in untreated stumps (*H*′ = 2.97 and 3.16, *D* = 0.52 and 0.57) (Table S1).

Dominance of single species, to some extent a converse of evenness, was greater in: (i) May than in July–August (*d* = 0.32 and 0.20); (ii) stumps < 5 cm diameter than in stumps > 5 cm diameter (*d* = 0.29 and 0.23); (iii) glyphosate-treated than in untreated stumps (*d* = 0.32 and 0.19). Differences between indices were often small.

There was more similarity between fungal communities (C_N_) in: (i) May than in July–August; (ii) stumps > 5 cm diameter than in stumps < 5 cm diameter; (iii) glyphosate-treated than in untreated stumps (Table 2).

## 3. Discussion

More than 250 fungal taxa were recorded from *P. serotina* stump wood (Table 1). The majority of taxa are ubiquitous and cosmopolitan. Many of those recorded have been isolated previously from roots [30], branches and dead wood of *P. serotina* in Poland [27].

The fungi were predominantly Ascomycota. Their high frequency is associated with initial decomposition of wood in this condition (decay class 1). Ascomycota are considered to be initial colonizers of dead hardwood [31]. Ascomycota generally cause soft wood rot (predominantly in wood that has an unusually high level of moisture), when cellulose and hemicellulose are degraded but more stable lignin is not [32]. Ascomycota colonize wood of deciduous species more readily than coniferous wood since it has more cellulose and hemicellulose [31,33].

The surface of wood is degraded by Ascomycota if the wood becomes repeatedly wet and dry, and exposed to high and low temperatures and to direct sunlight. The degradation causes checking of the surface, splitting and wood cell erosion. The moisture content of such wood is 40–220%. Decomposition is relatively slow and restricted to the surface layers (2–4 mm). In dry periods, wood crumbles, exposing the deeper layers, which are colonized by fungal mycelium and degraded during the subsequent wet period. Fungi colonize mainly exposed wood layers where concentrations of harmful carbon monoxide (CO) are lower. The effects of fungi and variable climatic conditions on wood decay and degradation of tissues become extensive in the long term. Wood of *P. serotina* is also cleavable and susceptible to crumbling and splitting [34].

Basidiomycota were less frequent. Generally they cause white or brown wood rot. During white wood rot, all wood components (cellulose, hemicellulose and lignin) are generally broken down simultaneously. On colonized surfaces, many Basidiomycota species belonging to subdivision Agaromycotina (from e.g., *Ceratobasidium* spp., *Colacogloea* spp., *Postia rennyi*, *Resinicium bicolor*, *Sistotrema brinkmanii*) or pileate, flabellate or cyphelloid (e.g., *Mycena* spp., *Schizophyllum* sp.) fruit bodies, with basidiospores formed on the surface (or underside) of the pileus, from which they are readily released to the air. Some produce structures that facilitate their spread and survival: *Resinicium bicolor* produces mycelial strands that are differentiated translocatory organs [35], which help to translocate chemical elements and secrete them in newly advancing zones of the mycelium in colonized and decaying wood.

The fungi recorded are frequently dark-colored (e.g., *Mollisia*, *Phialocephala*) and saturated with melanins, which are natural pigments and powerful antioxidants that contribute to virulence by interfering with host defense factors, including neutralizing the oxidative burst of phagocytic cells. They increase resistance of fungi, allowing tolerance of harsh and hostile environments, facilitating their survival and functioning, and helping in colonization and suppression of the plant-host defenses. They protect fungi against the effects of adverse temperatures, drought, heavy metals and radiation, whilst also neutralizing the effects of radicals formed under the influence of environmental stress [36,37]. By suppressing plant-host defense responses dark-pigmented fungi may help colonization of plants by other fungi, including pathogens. The pathogen *Chondrostereum purpureum* was not detected in the stump samples.

Many authors have reported that the different fungi found in the *P. serotina* stumps occur commonly on decomposing leaves, wood and bark of trees, as well as in many other habitats worldwide [38,39,40,41,42,43,44,45,46,47,48,49]. Extensive sexual and asexual reproduction guarantees their survival, allocation and distribution. Both sexual and asexual forms are adapted to different habitats within their area of distribution. The sexual stages occur in natural environments that are often highly unpredictable. Asexual forms are also common in anthropic environments.

Fungal communities in wood can be the source of active agents for new mycoherbicides. Attention should be paid to taxa that are saprotrophic, non-pathogenic on plants, animals or humans, frequent and cosmopolitan, tolerant of harsh environments and highly competitive. Such requirements are fulfilled by a few of the taxa recorded, including *Dictyochae* spp., *Mariannaea elegans*, *Mollisia* sp., *Phalocephala* sp., *Proliferodiscus* sp. (Ascomycota) and *Caratobasidium* spp., *Colacogloea* spp., *Mycena* spp., *Postia rennyi*, *Resinicium bicolor*, *Schizophyllum* sp., and *Sistotrema brinkmanii* (Basidiomycota).

There are, however, some limitations. Some species, before colonization, require appropriate preparation of wood. *Mycena* and *Sistotrema brinkmanii*, for example, are ’late colonizers’ and need prior decomposition of wood, usually by other white rot fungi. They may be effective after earlier treatment with another herbicide or mycoherbicide.

Biological control is always dependent on appropriate preparation and application of inoculum. In practice, preparation of inoculum of the fungi listed above should be relatively easy, since these fungi grow very well on artificial media and produce abundant, light, delicate but durable spores, guaranteeing effective introduction and further distribution in the habitat [38,50]. The use of specific media is sometimes necessary and recommended. In the case of *Mollisia*, adding a sour cherry extract to the in vitro culture medium proved advantageous [51]. In the case of *Phialocephala*, the addition of sawdust as a source of carbon is necessary [52]. Fungi originating from wood may always be cultured on sawdust, for example *Phlebiopsis gigantea* (Fr.) Jülich, used to prevent infection and colonization of conifer stumps by *Heterobasidion* spp.

For the safety of the environment and end users, mycoherbicides should not include phytopathogenic taxa, e.g., from the genera *Coniochaeta*, *Diaporthe* or *Laetiporus*. *Pleurophoma ossicola* may also present a risk because of its highly unusual origin. It had been recorded only from a bone lying under a young *Pinus sylvestris* L. tree in Germany [53]. This record seems to suggest that its identification in *P. serotina* wood in Poland could be a taxonomic error. Caution is advised also while selecting species from the genus *Ceratobasidium*. In our study it was identified somewhat imprecisely only to genus level. This is not especially informative, since the genus includes, apart from non-pathogenic species known from wood, also some phytopathogenic species, e.g., *Ceratobasidium cereale* D.I. Murray & Burpee, a causative agent of sharp eyespot (*Rhizoctonia*) in cereals which could be the source of pathogenic inoculum.

Caution is also advised when working with *R. bicolor* and *Schizophyllum* species. *Resinicium bicolor* is a pathogen of Douglas fir (*Pseudotsuga menziesii* (Mirb.) Franco) [54] and its introduction into areas where this tree grows is not recommended. *Schizophyllum commune* Fr. colonizes and destroys film-wrapped hay and silage bales [55], and thus should not be used in areas adjacent to agricultural areas, especially meadows.

The high frequency of *Penicillium* results only partly from its food preferences and ecological specialization. It is more often a consequence of abundant sporulation, the character of spores and their effective dispersal. The ubiquitous presence of *Penicillium* spp. in combination with their cellulolytic properties [56,57,58] suggests that they certainly participate in the decomposition of wood components. The scale of the process is probably limited and they should not be considered among the main active agents of potentially new mycoherbicides. They may, however, determine the efficacy and success of a main agent. They produce many mycotoxic metabolites, which may stimulate or inhibit the action of a mycoherbicide [59].

Glyphosate is a total (non-selective) herbicide. Although it is not a fungicide, we observed an absence of a few fungal taxa, i.e., *Candida mycetangii*, *Discosia pseudoartocreas*, *Mariannaea elegans*, *Pseudovalsaria ferruginea*, *Hyphoderma setigerum*, *Hypholoma acutum*, *Inocybe* sp., *Mycena galericulata* and *Postia rennyi*, after its application. Therefore, the above research should continue to confirm this trend.

Components of the glyphosate formulation facilitate penetration through cell membranes. Since the formulation affected the abundance and diversity of fungal communities in *P. serotina* wood, it is assumed that it also penetrates microbial membranes and may exhibit fungicidal action. Its time-dependent effect, with differences between spring and summer samples, may result from its response to weather conditions (higher temperature but lower moisture in summer). In spring, 8 months after application, fungal colonization of wood was not reduced (and may even have been promoted), whilst in summer, 10 months after application, it was reduced. A late, residual effect is frequently observed and typical of herbicides, which is to ’prevent the occurrence of weeds at the beginning of the next vegetation season’. Its activity may therefore be ’delayed’. Glyphosate, the active herbicide ingredient of Glifocyd 360 SL, is degraded by enzymes of soil microorganisms only [60] and is known for its high chemical stability. Its degradation products include sarcosine, aminomethylphosphonic acid (AMPA), ammonia, ethanol, water and phosphates. AMPA may be accumulated or degraded at an even slower rate than glyphosate. It leads to a late fungicidal effect. An initial increase in fungal abundance due to the slow degradation and accumulation of AMPA has also been reported [60,61,62]. Other compounds produced during glyphosate degradation (e.g., ethanol, sarcosine) are bactericidal and fungicidal. They may contribute to the final fungicidal effect. Sarcosine, which is an amino acid, and ammonia, alkalize the habitat and reduce the abundance of fungi, which prefer acidic conditions. It is also likely that temperature contributes to the final effect. The document GLIFOCYD 360 SL, Permit of the Ministry of Agriculture and Rural Development no. R–94/2017 of 24 May 2017 indicates that ’high temperature and humidity as well as strong insolation accelerate its weed killing action’.

## 4. Materials and Methods

Black cherry trees (*P. serotina*), 10–20 years old, grown in Podanin Forest District (19°28′00″ E 52°04′00″ N), were felled in 2015 leaving stumps 15 cm high. In August 2016, the stumps were treated by spraying directly with glyphosate, isopropylamine salt (Glifocyd 360 SL) at 6 L ha^−1^. Untreated control stumps were sprayed with water.

In May and July–August 2017, 48 wood discs, 2 cm thick, were collected from the surface of the 2-year-old stumps (24 discs from six glyphosate-treated stumps and 24 from six control stumps). Eight experimental variants (2 herbicide treatments × 2 sample times × 2 disc diameters) were compared. Variants were: (1) May, <5 cm diameter, treated with glyphosate; (2) May, >5 cm diameter, treated; (3) May, <5 cm diameter, untreated; (4) May, >5 cm diameter, untreated; (5) July–August, <5 cm diameter, treated; (6) July–August, >5 cm diameter, treated; (7) July–August, <5 cm diameter, untreated; (8) July–August, >5 cm diameter, untreated. Each variant had three sub-samples. Stump diameters did not exceed 15 cm. Stumps were located at a minimum of 5 m apart. Stump wood was in decay class 1 [63], treated stumps with glyphosate were dead, but the living shoots were observed in untreated stumps. The mean temperature was 13.8 °C in May and 18.2 °C in July–August.

In the laboratory, sub-samples of sawdust were taken from each disc using a cordless SPARKY BUR2 15E drill. Sub-samples were pooled and ground in a SPEXTM SamplePrepTM Freezer/MillTM cryogenic mill. Environmental DNA was extracted with Plant Genomic DNA Purification Kit (Thermo Fisher Scientific). The ITS1, 5.8S rDNA region was used to identify the fungal species, and the analysis was carried out with primers ITS1FI2 5′-GAACCWGCGGARGGATCA-3′ [64] and 5.8S 5′-CGCTGCGTT CTTCATCG-3′ [65]. Each amplification reaction was carried out in a final volume of 25.0 μL containing 2 μL DNA, 0.2 μL of each primer, 10.1 μL deionized water and 12.5 μL 2× PCR MIX (A & A Biotechnology, Gdynia, Poland). Initial denaturation was at 94 °C for 5 min. Cycling conditions were: Primer annealing at 56 °C for 30 s, elongation at 72 °C for 30 s and final elongation at 72 °C for 7 min, with 35 cycles. Visualization of 5 μL amplicons was performed in 1.0% agarose gel dyed with Midori Green Advance DNA (Genetics). The PCR products were purified with MinElute PCR Purification Kit (Qiagen). The amplicons were sequenced using the Illumina system in the Genomic Laboratory, DNA Research Center, Rubież 46, Poznań, Poland. Synthetic mock community and negative (no DNA added) controls were included to assess sequencing quality and removed the OTUs present in the control with only one-two sequences. The results were subjected to bioinformatic analysis [66]. Sequences were compared with already deposited sequences in the GenBank^®^ of the National Center for Biotechnology Information (NCBI) of the U.S. National Library of Medicine using BLASTn 2.8.0 (Nucleotide Basic Local Alignment Search Tool) [67,68,69,70], considering a similarity cutoff of 97%. The BLAST search excluded sequences from model (XM/XP) and uncultured and environmental samples. An additional BLAST search, with 85% similarity cutoff, was conducted considering only deposited sequences from type material. Identification was made to the rank of the lowest taxon. Abundance of fungi was defined as the number of OTUs in a sample. Frequency of an individual taxon was defined as percentage (%) of OTUs in the total number of OTUs. Diversity was defined as the number of species in a sample. The role of the fungal species detected in the community was determined on the basis of published work.

### Statistical Analysis

Differences between variants in numbers of OTUs and taxa were analyzed using the chi-squared test (χ^2^). Diversity in microbial communities was compared with diversity indices calculated for each community, including their abundance and taxonomic composition [71]. Diversity in a community was indicated by Margalef’s diversity index (*D*_Mg_), based on species richness, and Shannon’s diversity index (*H*′), which considers species richness and evenness. Evenness and dominance were indicated by Simpson’s diversity index (*D*), Shannon’s evenness index (*E*) and Berger–Parker’s dominance index (*d*). The similarity between fungal communities was determined by calculating Sorensen’s qualitative similarity index (C_N_). Similarity and relationships among fungal communities are shown by a heat map.

## 5. Conclusions

Herbicides may be selectively fungicidal. They affect the abundance and diversity of fungal communities in wood of deciduous trees. Greater frequency of Ascomycota in herbicide-treated stumps indicates more tolerance of such toxic substances in Ascomycota.

## Figures and Tables

**Table 1:** Fungi in stump wood of *P. serotina* in relation to sample time, stump diameter and glyphosate treatment.

Taxon	Frequency in the Fungal Community (%)							
	May Samples				July–August Samples			
	<5 cm Diam.		>5 cm Diam.		<5 cm Diam.		>5 cm Diam.	
	Treated	Untreated	Treated	Untreated	Treated	Untreated	Treated	Untreated
Blastocladiomycota	0.046	0	0	0	0	0.097	0	0
Rozellomycota	0	0.164	0	0.017	0.019	0	0.049	0.672
Glomeromycota	0	0.25	0	0.02	0.02	0	0.05	0.67
Zygomycota	0.023	0.62	0.34	0.49	0.02	0.02	0.01	0.09
Ascomycota	74.51	54.85	54.45	56.25	39.15	58.25	48.4	44.62
Basidiomycota	14.8	11.76	5.30	21.93	30.22	34.26	40.11	48.32
Other taxa	3.96	0.58	13.38	2.53	4.77	2.57	3.61	2.65
Non-cultured fungi	3.65	12.75	21.0	5.81	21.75	3.16	4.86	2.45
Organisms with no NCBI reference sequence	3.01	19.2	5.49	12.98	4.07	1.65	2.96	1.24
Number of Operational Taxonomic Units	20,198				38,770			
	6756		13,442		14,585		24,185	
	4324 ^a^	2432 ^a^	7673 ^b^	5769 ^b^	5282 ^c^	9303 ^c^	8110 ^d^	16,075 ^d^
Number of fungal Operational Taxonomic Units	17,073				36,758			
	5974		11,099		13,726		23,032	
	4023 ^a^	1951 ^a^	6225^b^	4874 ^b^	4815 ^c^	8911 ^c^	7577 ^d^	15,455 ^d^
Number of taxa	285 ^a^	120 ^a^	242 ^b^	304 ^b^	267	287	319	306
Number of fungal taxa	249 ^a^	83 ^a^	217 ^b^	281 ^b^	229	254	277	274

^a–d^—the same letter indicates a statistically significant difference between treatments in each pairwise comparison according to χ^2^ test, *p* ≤ 0.001 or *p* ≤ 0.05.

**Table 2:** Sørensen coefficients of similarity (C_N_) for comparing fungal communities from different stump sizes and with or without glyphosate treatment.

May				July–August			
0.8892							
<5 cm diam.				>5 cm diam.			
0.8419				0.7904			
<5 cm diam.		>5 cm diam.		<5 cm diam.		>5 cm diam.	
Treated	Untreated	Treated	Untreated	Treated	Untreated	Treated	Untreated
0.22		0.44		0.46		0.53	

## Supplementary Materials

The following is available online at https://www.mdpi.com/2223-7747/9/9/1126/s1, Table S1: Taxa of fungi occurring in black cherry stumps in relation to sample time, stump diameter and glyphosate treatment.
