# Supplementary material for: Communities of Fungi in Black Cherry Stumps and Effects of Herbicide
Source: Plants (Basel). 2020 Aug 31;9(9):1126. doi: 10.3390/plants9091126 (PMC7570310; doi:10.3390/plants9091126)

Table S1. Taxa of fungi occurring in black cherry stumps in relation to sample time, stump diameter and glyphosate treatment.

**Taxon**	**Order**	Similarity of the reference sequence	Frequency in the fungal community (%)							
			May stumps				July–August stumps			
			< 5 cm diam.		> 5 cm diam.		< 5 cm diam.		> 5 cm diam.	
			Treated	Untreated	Treated	Untreated	Treated	Untreated	Treated	Untreated
**Glomeromycota**										
Glomeraceae sp.	Glomerales	99%	0	0.082	0	0	0	0	0	0
**Zygomycota**										
Absidia cylindrospora	Mucorales	98%	0	0	0	0	0	0.011	0	0
Mortierella hyalina	Mortierellales	100%	0	0.37	0	0	0	0	0	0
Mortierellaceae	Mortierellales	97%	0	0	0.013	0	0	0.011	0.012	0
Mucor bainieri	Mucorales	99%	0	0	0.235	0	0	0	0	0
Umbelopsis isabellina	Umbelopsidales	100%	0.023	0.247	0.091	0.485	0.019	0	0	0.087
**Ascomycota**										
Absconditella sp.	Ostropales	98%	0.023	0	0.013	0.017	0.019	0.021	0	0
Acephala applanata	Helotiales	100%	0	0.123	0	0	0	0	1.023	0.056
Acremonium fusidioides + *Acremonium* spp.	Hypocreales	99–100%	0.023	0	0.339	0	0	0.064	0	0.068
Adisciso tricellulare	Xylariales	97%	0.046	0	0.013	0	0.038	0.129	0	0.006
Agonimia sp.	Verrucariales	98%	0.069	0	0.013	0	0.114	0.441	0	0
Alatospora acuminate + *Alatospora* sp.	Leotiales	99–100%	0	0	0.013	0.017	0	0	0	0.043
Arthrobotrys foliicola	Orbiliales	99%	0	0	0.091	0	0.038	0.032	0	0
Articulospora sp.	Orbiliales	99%	0	0	0	0.017	0.398	0.527	0.062	0.124
Ascomycota		100%	3.84	12.58	35.32	16.00	4.26	2.43	22.18	2.56
Barbatosphaeria arboricola + *B. barbirostris* +*B. dryina* + *B. neglecta*	Incertae sedis	97–99%	0.092	0	1.134	0	0.038	0	0.062	0.647
Barssia maroccana	Pezizales	98%	0	0.123	0	0	0	0	0.469	0
Beauveria pseudobassiana	Hypocreales	100%	0.116	0.082	0	0.104	0.057	0.247	0.025	0.006
Biatora sphaeroidiza	Lecanorales	100%	0	0	0	1.369	0	0.011	0.16	0.411
Bionectriaceae	Hypocreales	100%	0.023	0	0	0.017	0	0	0	0
Blastobotrys sp.	Saccharomycetales	100%	0.046	2.015	0	0	0	0	0	0
Cadophora finlandica + *C. gregat* + *C. luteo-olivacea* + *C. melinii* + *Cadophora* sp.	Helotiales	99–100%	0.138	0	0	0.087	0.266	0.161	0.234	2.357
Caliciopsis beckhausii	Coryneliales	99%	0	0	0.065	0.052	0	0	0	0
Candida fructus + *C. mycetangii* + *C. sake* + *Candida* sp.	Saccharomycetales	99–100%	0.092	1.974	0	1.473	0.076	0	0.049	0.006
Capronia epimyces + *C. pilosella*+ *C. pulcherrima* + *Capronia* sp.	Chaetothyriales	99–100%	0.763	0	0.743	0.087	0.076	0	0.296	0.137
Cephaliophora longispora	Pezizales	97%	0	0	0	0	0	0	0.16	0
Cephalosporium spp.	Pezizales	98%	0.046	0	0.013	0.017	0.151	0	0.148	0.006

Taxon	Order	Similarity of the reference sequence	Frequency in the fungal community (%)							
			May stumps				July–August stumps			
			< 5 cm diam.		> 5 cm diam.		< 5 cm diam.		> 5 cm diam.	
			Treated	Untreated	Treated	Untreated	Treated	Untreated	Treated	Untreated
Cephalothecaceae	Sordariales	98%	0.578	0.082	0.482	0.503	0	0.107	0.074	0.019
Chaetomium sp.	Sordariales	98%	0.023	0	0	0.017	0.095	0	0	0
Chaetosphaeria myriocarpa + *Ch. rivularia*	Chaetosphaeriales	98–99%	0.023	0	0	0	0	0.021	0.049	0.05
Chaetothyriales	Chaetothyriales	100%	2.035	0	0.678	0.589	1.003	1.172	0.863	0.1
Chalara pseudoaffinis + *Chalara* sp.	Microascales	99–100%	0	0.041	0	0.017	0.928	0.086	0	0
Chloridium sp.	Chaetosphaeriales	100%	0.023	0.123	0	0	0	0	0	0
Ciborinia candolleana	Helotiales	98%	0	0.041	0	0.017	0.038	0.107	0.012	0.006
Ciliophora sp.	Incertae sedis	100%	0.116	0.123	0.365	0	0.189	0.011	0	0
Cistella sp.	Helotiales	100%	0	0	0.013	0	0	0	0	0
Cladobotryum paravirescens	Hypocreales	100%	0.023	0	0	0	0	0	0	0
Cladophialophora sp. + *C. chaetospira* + *C. arxii*	Chaetothyriales	98–100%	1.087	0	0.182	0.364	0.852	0.656	0.925	0.46
Claussenomyces sp.	Phacidiales	99%	0	0	0.026	0.017	0	0	0.111	0
Colletotrichum antirrhinicola + *C. godetiae* + *C. tamarilloi*	Incertae sedis	99–100%	0.092	0	0	0	0.435	1.763	0.024	0.006
Collophora sp. + *C. paarla*	Incertae sedis	98–99%	0.023	0	0.078	0.104	0.076	0	0.308	0.069
Colpoma quercinum	Rhytismatales	98%	0	0	0	0.017	0	0	0	0
Coniochaeta sp. + *C. lignicola* + *C. fodinicola*	Coniochaetales	98–99%	0.717	0	2.854	0.121	0.189	0.011	0.358	0.33
Coniochaetaceae	Coniochaetales	98%	0	0	0	0	0	0	0.012	0
Coniochaetales	Coniochaetales	97%	0.023	0	0.013	0	0	0.011	0.025	0.012
Crocicreas epicalamia + *Crocicreas* sp.	Helotiales	99–100%	0.023	0.206	0.013	0.017	0.038	5.493	0.481	0.025
Cyphellophora reptans	Chaetothyriales	99%	0	0	0.013	0.191	0	0	0	0
Dendrophoma cytisporoides	Incertae sedis	100%	0.023	0	0	0	0	1.666	0	0
Dermateaceae	Helotiales	99%	0.023	0.206	0.013	0.052	0	0.161	0	0
Desertella sp.	Incertae sedis	100%	0.023	0	0.065	0.156	0.057	0.247	0.012	0.006
Diaporthe cotoneastri + *D. cynaroidis* + *D. helicis*	Diaporthales	99–100%	0.139	0	0.013	0.035	6.039	17.274	0.173	0.087
Dictyochaeta sp.	Chaetosphaeriales	99%	0	0.164	0	0.017	0.019	13.834	0	0.1
Discosia pseudoartocreas	Incertae sedis	99%	0.069	0	0	0.069	0	0.484	0	0
Discostroma sp.	Xylariales	99%	0	0	0	0.069	0	0.011	0	0
Exophiala bergeri + *E. capensis* + *E. castellanii* + *E. lecanii-corni* + *E. opportunistica* + *E. sideris* + *E. psychrophila* + *Exophiala* sp.	Chaetothyriales	99–100%	0.947	0	1.121	1.005	0.493	0.398	0.999	0.859
Fusarium oxysporum + *F. cyanostomum* + *Fusarium* sp.	Hypocreales	99–100%	0.046	0	0.013	0.104	0.057	0.032	0.024	0
Fusicladium cordae	Pleosporales	100%	0	0	0	0.017	0	0	0	0
Geomyces auratus	Helotiales	98%	0	0	0	0.035	0.019	0	0	0
Gnomoniopsis paraclavulata	Diaporthales	100%	0.023	0	0	0	0	0.075	0	0.006
Haloguignardia irritans	Lulworthiales	100%	0	0	0	0	0.909	0	0	0

Taxon	Order	Similarity of the reference sequence	Frequency in the fungal community (%)							
			May stumps				July–August stumps			
			< 5 cm diam.		> 5 cm diam.		< 5 cm diam.		> 5 cm diam.	
			Treated	Untreated	Treated	Untreated	Treated	Untreated	Treated	Untreated
Halosphaeriaceae	Microascales	100%	0	0	0.013	0	0	0	0.025	0
Harzia cameroonensis	Melanosporales	100%	0	0	0	0	0	0	0	0.062
Helotiaceae	Helotiales	100%	0	0	0	0.624	0.019	0	0.062	0
Helotiales	Helotiales	100%	0.532	1.192	0.235	0.312	0.492	0.623	4.242	0.74
Herpotrichiellaceae	Chaetothyriales	99%	0.856	0.041	1.277	2.704	0.398	1.752	0.37	0.51
Humicola nigrescens + *Humicola* sp.	Sordariales	99–100%	0.023	0	0	0.416	0	0	0.012	0
Hyalorbilia inflatula	Helotiales	99%	0	0	0.026	0.017	0	0	0	0.012
Hyaloscyphaceae	Helotiales	99%	0.046	0	0	0.035	0	0	0	0.012
Hydnotrya tulasnei	Pezizales	99%	0	0.041	0	0	0	0	0	0
Hyphodiscus hymeniophilus	Helotiales	99%	0	0	0	0.052	0	0	0.012	0.006
Hypocreales	Hypocreales	99%	0	0.164	0.091	0.537	0.36	0.064	0.012	0.019
Hypogymnia physodes	Lecanorales	99%	0	0	0	0	0	0	0	0.124
Hypomyces lactifluorum	Hypocreales	99%	0	0	0	0.017	0.019	0.011	0.012	0
Ilyonectria mors-panacis + *I. robusta*	Hypocreales	99–100%	0	0	0	0	0	0.011	0.012	0.012
Infundichalara minuta	Helotiales	100%	0.023	0.206	0.039	0.052	1.25	0.011	0.062	0.019
Ionomidotis irregularis	Helotiales	100%	0	0	0	0	0	0.204	0.012	0
Jattaea aphanospora + *J. prunicola* + *J. ribicola*	Calosphaeriales	99–100%	0.647	0.041	1.942	0.139	0	0.011	0.629	0.018
Junewangia queenslandica	Incertae sedis	98%	0.301	0	0.013	0.069	0	0.011	0	0.585
Kohlmeyeriopsis medullaris	Magnaporthales	97%	0.069	0	0	0	0.019	0	0.012	0
Lachnellula calyciformis	Helotiales	99%	0.278	0.411	0	0.156	0.076	0	0.012	0.006
Lachnum pulverulentum + *L. virgineum*	Helotiales	99–100%	0.162	0	0.013	0	0	0	0.025	0.012
Lasiosphaeria rugulosa	Sordariales	99%	0	0	0	0	0	0.011	0.025	0.006
Lasiosphaeriaceae sp.	Sordariales	99%	0	0	0	0	0	0	0	0.062
Lecania sp.	Lecanorales	99%	0.023	0	0.013	0.017	0	0	0.086	0
Lecanicillium muscarium	Hypocreales	100%	0	0	0	0.035	0.019	0	0.012	0
Lecanorales	Lecanorales	100%	0.023	0	0.013	0.017	0.076	0.129	0.025	0
Lecanoromycetes		100%	0.185	0	0.417	0.329	0.057	2.236	0.234	0.006
Lecophagus sp.	Pezizales	100%	0.023	0	0	0.347	0	0.011	0	0
Leotiomycetes		100%	0.324	0.082	1.564	0.988	1.59	0.172	1.911	6.432
Lepraria elobata + *L. incana*	Lecanorales	99–100%	0	0	0	0.069	0.057	0	0.012	0.044
Leptodontidium trabinellum	Incertae sedis	100%	0.046	0	0	0.329	0	0	0.012	0.902
Lophium arboricola	Mytilinidiales	100%	0.023	0	0	0.052	0.057	0.011	0	0.025
Lophodermium pinastri	Rhytismatales	100%	0	0.452	0	0	0	0	0	0
Mariannaea elegans	Hypocreales	99%	0	0	0	0	0	0	0	8.585

Taxon	Order	Similarity of the reference sequence	Frequency in the fungal community (%)							
			May stumps				July–August stumps			
			< 5 cm diam.		> 5 cm diam.		< 5 cm diam.		> 5 cm diam.	
			Treated	Untreated	Treated	Untreated	Treated	Untreated	Treated	Untreated
Massaria parva	Pleosporales	99%	0	0	0	0	0	0	0	0.019
Meliniomyces sp.	Incertae sedis	99%	0	0	0.104	0	0	0	0.037	0.367
Meliniomyces variabilis	Incertae sedis	99%	0	0	0.013	0	0	0.011	0	0.019
Menispora ciliata + *M. manitobaensis*	Chaetosphaeriales	99–100%	0.023	0	0	0.156	0.038	0.064	0.012	0.006
Metapochonia bulbillosa	Hypocreales	99%	0	0	0	0.087	0.038	0	0.037	0.012
Micarea assimilata + *M. leprosula*	Lecanorales	99–100%	0.162	0	0.117	0.017	0.095	0.075	0.037	0
Mollisia cinerea + *Mollisia* sp.	Helotiales	99–100%	52.27	0.00	0.51	0.02	1.59	0.00	0.76	3.41
Mycofalcella calcarata	Incertae sedis	100%	0.023	0	0	0	0	0	0	0
Mycoleptodiscus sp.	Magnaporthales	100%	0	0	0.013	0.035	0.038	0.118	0.025	0.031
Myrothecium sp.	Hypocreales	100%	0	0	0	0	0	0.011	0	0
Myxotrichaceae	Onygenales	100%	0	0	0	0	0	0.021	0	0.006
Nakazawaea ernobii	Saccharomycetales	99%	0.139	0	0	0	0	0	0.321	0.037
Nectria cinnabarina	Hypocreales	99%	0	0	0	0	0	0.011	0	0.012
Nectriaceae	Hypocreales	99%	0.185	0	0.104	0.052	1.344	0	0.234	0.075
Neobulgaria pura + *Neobulgaria* sp.	Helotiales	99–100%	0.162	0	0.039	0	0	0.054	0.024	0.019
Neofabraea sp.	Helotiales	99%	0	0	0.013	0.087	0.019	0	0	0.006
Nothojafnea cryptotricha	Pezizales	99%	0	0	0	0	0	0.011	0	0
Oidiodendron majus + *O. periconioides*	Incertae sedis	98-99%	0	0	0	0.017	0	0	0.185	0.019
Onygenaceae	Onygenales	100%	0.023	0	0	0.052	0.038	0.064	0.012	0.025
Ophiostoma tsotsi	Ophiostomatales	99%	0	0	0	0.069	0	0	0.012	0.012
Ophiostomataceae	Ophiostomatales	99%	0	0	0	0.087	0	0.011	1.122	0.006
Orbilia aprilis + *O. aristata*	Orbiliales	99–100%	0.023	0	0.482	0.156	0	0.053	0.037	0
Orbiliomycetes	Orbiliales	100%	0.023	0	0.326	0.052	0.038	0.054	0.062	0.031
Otidea subterranea	Pezizales	100%	0.278	0	0.078	0.069	0.057	0.097	0.222	0.112
Pannaria athroophylla	Peltigerales	99%	0	0	0	0.087	0	0	0	0
Parmelia subdivaricata	Lecanorales	99%	0.023	0	0.039	0.017	0.019	0.043	0.012	0
Penicillium angulare + *P. bialowiezense* + *P. cairnsense* + *P. citreonigrum* + *P. kongii* +*P. lanosum* + *P. lapidosum* + *P. miczynskii* + *P. raphiae* + *P. viticola*	Eurotiales	99–100%	0.763	5.345	0.182	1.143	0.322	0.784	0.962	1.107
Petriella sordida	Microascales	99%	0.046	0	0	0	0	0	0.025	0.025
Pezicula sporulosa	Helotiales	100%	0.116	0	0.078	0.191	0.265	0.032	0.567	0.056
Phacidium grevilleae	Phacidiales	100%	2.567	0	0.156	0.173	0.625	1.526	0.185	0.081
Phaeoacremonium hungaricum + *P. occidentale*	Togniniales	98–100%	0	0	0.013	0	0.019	0	0.271	0
Phaeomollisia piceae	Helotiales	100%	0.139	0	0.274	0.035	0.17	0.032	0.21	0.068
Phaeomoniella sp.	Phaeomoniellales	99%	0.185	0	0.3	0.017	0	0	0.16	0.05

Taxon	Order	Similarity of the reference sequence	Frequency in the fungal community (%)							
			May stumps				July–August stumps			
			< 5 cm diam.		> 5 cm diam.		< 5 cm diam.		> 5 cm diam.	
			Treated	Untreated	Treated	Untreated	Treated	Untreated	Treated	Untreated
Phialocephala compacta + *P. fusca.* + *P. glacialis* + *P. scopiformis* + *Phialocephala* sp.	Helotiales	99-100%	0.092	0.329	0.026	0.572	7.459	0.011	0.469	0.224
Phomatospora dinemasporium	Incertae sedis	100%	0.185	0	0	0	0.076	0.032	0.049	0
Phomopsis pulla (Sacc.)	Diaporthales	100%	0	0	0	0	0.246	0.075	0	0
Picoa juniperi	Pezizales	98%	0	0	0	0.676	0	0	0	0.006
Plectania melastoma	Pezizales	99%	0	0	0	0	2.934	0.032	0.012	0
Pleurophoma ossicola	Xylariales	99%	0.046	25.37	0.352	0.225	0.076	0.021	0.025	0.031
Proliferodiscus sp.	Helotiales	99%	0.185	0.411	0.261	14.751	0.341	0.828	1.48	5.636
Pseudeurotiaceae	Incertae sedis	97%	0	0	0	0.035	0	0	0	0
Pseudogymnoascus verrucosus	Incertae sedis	100%	0	0	0	0.451	0.057	0	0	0.006
Pseudovalsaria ferruginea	Boliniales	100%	0	0	0	0	0	0	0	0.821
Pyrenopeziza revincta	Helotiales	100%	0.093	0	0	0	0.095	0	0	0
Pyrenula septicollaris	Pyrenulales	100%	0	0	0	0	0	0	0	0.012
Rhizoscyphus sp.	Helotiales	100%	0.023	0	0.222	1.317	0.019	0	0	0
Saccharomycetales	Saccharomycetales	99%	0.093	0	0.013	0.26	0	0.011	0.419	4.871
Sarea resinae	Trapeliales	99%	0.116	0	0.013	0.069	0.114	0.107	0.037	0.006
Sarocladium strictum	Incertae sedis	99%	0.046	0	0	0.711	0.038	0.107	2.01	0.156
Scheffersomyces ergatensis	Saccharomycetales	99%	0.37	0	0	0	0	0	0	0
Sordariales	Sordariales	100%	0.023	0	0.143	0.017	0	0	0	0
Sordariomycetes	Sordariales	98%	0.069	0.082	0.443	0.416	1.174	0.054	0.197	0.174
Sporothrix dimorphospora + *S. pallida*	Ophiostomatales	99–100%	0	0	0	0.208	0.095	0	0.062	0.025
Stachybotrys sp.	Hypocreales	98%	0	0	0	0.26	0	0	0.012	0
Talaromyces amestolkiae + *T. verruculosus* + *T. wortmannii*	Eurotiales	98–100%	0	0.164	0	0.07	0	0	0	0.012
Taphrina carnea Johanson + *T. confusa*	Taphrinales	99–100%	0.185	0	0	0.035	0.057	0.032	0.012	0.025
Tetracladium setigerum	Helotiales	99%	0	0	0	0	0	0	0.012	0
Tolypocladium sp.	Hypocreales	99%	0	0	0.013	0.087	0	0.011	0.074	0.012
Trichoderma asperellum + *T. bissettii* + *T. crassum* + *T.stellatum* + *T. theobromicola*	Hypocreales	99–100%	0.184	0.37	0.339	0	0.019	0.043	0.012	0
Tridentaria implicans	Incertae sedis	99%	0	0	0.026	0.035	0	0.021	0.012	0.019
Trimmatostroma cordae	Helotiales	99%	0	0	0	0.017	0	0.075	0	0
Truncatella restionacearum	Xylariales	100%	0.023	0	0.078	0.087	0.038	0.032	0	0.012
Tumularia sp.	Incertae sedis	100%	0.069	0.082	0.195	2.236	0	0.441	0.025	0.025
Valsaceae	Diaporthales	100%	0.231	0.041	0.091	0.347	0.095	0	0.049	0
Valsaria lopadostomoides	Incertae sedis	99%	0.023	0	0	0	0	0	0.296	0.056

Taxon	Order	Similarity of the reference sequence	Frequency in the fungal community (%)							
			May stumps				July–August stumps			
			< 5 cm diam.		> 5 cm diam.		< 5 cm diam.		> 5 cm diam.	
			Treated	Untreated	Treated	Untreated	Treated	Untreated	Treated	Untreated
Venturia hystrioides + *Venturia* sp.	Venturiales	99–100%	0.023	0	0.026	0.156	0	0.011	0	0.025
Venturiaceae	Venturiales	99%	0	0	0	0.035	0.019	0.054	0.086	0
Venturiales	Venturiales		0.046	0	0.013	0.069	0	0.011	0	0
Xenopolyscytalum pinea	Helotiales	97%	0	0	0	0.017	0	0.011	0	0
Xylariaceae	Xylariales	100%	0.023	0.082	0	0	0	0.204	0	0
Yamadazyma mexicana	Saccharomycetales	99%	0.116	0	0	0.052	0.019	0	0.345	0
Yarrowia lipolytica	Saccharomycetales	99%	0.093	2.056	0	0	0.019	0	0	0
**Basidiomycota**										
Agaricaceae	Agaricales	100%	0.046	0.082	0.756	0.035	0.057	0.043	0.037	0.012
Agaricales		100%	2.845	0.164	0.052	0.052	2.385	2.311	1.196	0.398
Agaricomycetes		100%	0.069	0.041	0.013	0.017	0.019	0.032	0.999	0.691
Agaricostilbales		100%	0	0	0	0.017	0.114	0.064	0	0.006
Amanita parcivolvata	Agaricales	100%	0	0	0.013	0.017	0.095	0.054	0.049	0.025
Amylocorticiellum molle	Boletales	99%	0	0	0	0	0.587	0	0.025	0
Auriculariales		99%	0.023	0.041	0	0	0.019	1.666	0.012	0.019
Basidiomycota		99%	0.532	0.535	0.352	1.161	1.647	8.266	0.752	0.79
Bensingtonia sp.	Agaricostilbales	100%	0.046	0	0	0	0	0.011	0.049	0.019
Buckleyzyma aurantiaca + *B. phyllomatis*	Buckleyzymales	98–100%	0	0	0	0	0.019	0	0	0.006
Bullera melastomatis	Tremellales	99–100%	0.093	0	0	0.017	0	0.086	0	0.006
Bulleromyces albus	Tremellales	99%	0	0	0	0.017	0	0.011	0	0.031
Cantharellales	Cantharellales	100%	0	0.082	0.013	0	0.114	0.032	0.321	0.006
Ceratobasidiaceae	Cantharellales	100%	0.023	0	0	0	0.644	0	0.025	0
Ceratobasidium	Cantharellales	100%	0.023	0	0	0	0.019	0	0.469	11.944
Cheimonophyllum roseum	Agaricales	100%	0	0	0	0	0	0	0.222	0
Chionosphaera cuniculicola	Agaricostilbales	99%	0.948	0	0	0.017	0	0	0.037	0
Chrysozymaceae		100%	0.023	0	0	0.017	0.322	0	0.21	0.012
Clavaria neonigrita	Agaricales	99%	0	0	0	0	2.12	0	0	0
Clitopilus hobsonii	Agaricales	99%	0	0	0	0.052	0	0	0	0
Colacogloea peniophorae + *C. philyla* + *Colacogloea* sp.	Heterogastridiales	99–100%	2.036	0	0.065	0.139	0.246	0.054	4.18	0.062
Conocybe echinata	Agaricales	100%	0	0	0	0	0.019	0	0	0
Corticium confine	Corticiales	100%	0	0	0	0.017	0	0	0	0
Cryptococcus pseudolongus + *C. psychrotolerans* + *Cryptococcus* sp.	Tremellales	99–100%	2.197	0.041	0.052	0.641	0.853	2.278	0.419	0.118
Cuniculitrema polymorpha	Tremellales	98%	0.023	0	0	1.837	2.367	0	12.528	2.519
Curvibasidium cygneicollum	Microbotryales	100%	0.162	0	0.678	1.612	0.454	0.14	0.086	0.199

Taxon	Order	Similarity of the reference sequence	Frequency in the fungal community (%)							
			May stumps				July–August stumps			
			< 5 cm diam.		> 5 cm diam.		< 5 cm diam.		> 5 cm diam.	
			Treated	Untreated	Treated	Untreated	Treated	Untreated	Treated	Untreated
Cystobasidiomycetes		100%	0	0	0.169	0.069	0.076	0.032	0.012	0.037
Cystobasidium pinicola	Cystobasidiales	100%	0.301	0	0.13	0.537	0.133	0.161	0.444	0.485
Cystofilobasidiales	Cystofilobasidiales	100%	0.046	0	0	0.173	0	0.043	0	0.025
Cystofilobasidium infirmominiatum + *C. macerans*	Cystofilobasidiales	99–100%	0.069	0	0.013	0.069	0	0.011	0.012	0.012
Dacrymyces chrysospermus	Dacrymycetales	99%	0	0	0	0.485	0	0.032	0	0
Dioszegia changbaiensis + *D. fristingensis*	Tremellales	98–99%	0	0	0.039	0.017	0	0	0	0
Erythrobasidiales	Erythrobasidiales	100%	0.093	0	0	0.069	0.076	0.29	0.012	0.019
Erythrobasidium hasegawae + *Erythrobasidium* sp.	Erythrobasidiales	99–100%	0.046	0	0.013	0.052	0.227	0.075	0.012	0.006
Exobasidium arescens + *E. kishianum* + *E. maculosum* + *Exobasidium* sp.	Exobasidiales	99–100%	0.069	0	0.039	0.624	0.038	0.129	0.974	0.062
Fellomyces sp. + *F. borneensis* + *F. horovitziae* + *F. mexicanus*	Tremellales	99–100%	0.232	0	0.039	0.26	0.152	0.538	0.11	0.043
Fellozyma inositophila	Incertae sedis	99%	0.093	0	0.143	1.231	0.019	0.011	0.123	0.031
Fibulobasidium inconspicuum + *F. murrhardtense*	Tremellales	98–99%	0.023	0	0.209	0.035	0.019	0	0	0.024
Filobasidium stepposum + *F. wieringae*	Filobasidiales	99–100%	0.023	0	0.013	0.087	0	0	0.049	0.019
Flammulina velutipes	Agaricales	100%	0	0	0	0	0	0	0	0.062
Genolevuria amylolytica + *G. bromeliarum*	Tremellales	99–100%	0.046	0	0	0.017	0	0.161	0	0.012
Hamamotoa lignophila	Incertae sedis	99%	0.023	0	0.039	1.04	0.057	0.011	0.567	1.033
Hydnaceae	Cantharellales	100%	0	0.082	0	0	0	0.011	0	0
Hygrocybe subceracea	Agaricales	100%	0	0	0	0	0.133	0.021	0	0
Hygrophoraceae	Agaricales	100%	0	0.082	0	0.017	0	0	0	0
Hymenochaetales	Hymenochaetales	100%	0	0.123	0	0	0	0	0	0
Hyphoderma setigerum	Polyporales	99%	0	0	0	0	0	0	0	0.747
Hypholoma acutum	Agaricales	99%	0	0	0	0	0	0	0	0.292
Inocybe sp.	Agaricales	100%	0	0	0	0.884	0	0	0	0
Itersonilia pannonica + *I. perplexans*	Cystofilobasidiales	99–100%	0.023	0	0.026	0.156	0.076	0.215	0	0.037
Kockovaella machilophila	Tremellales	99%	0.046	0	0.117	0.676	0	0.054	0.136	0.056
Kondoa aeria	Agaricostilbales	99%	0	0	0	0.017	0	0.011	0	0.037
Krasilnikovozyma huempii	Cystofilobasidiales	99%	0	0	0	0	0	0	0.012	0.05
Kriegeria eriophori	Kriegeriales	99%	0	0	0	0.156	0	0	0	0
Kurtzmanomyces sp. + *K. insolitus* + *K. tardus*	Agaricostilbales	99–100%	0.069	0	0.013	0.035	0.246	0.139	0.074	0
Kwoniella pini + *K. shivajii*	Tremellales	98–99%	0.162	0	0.039	0.139	0.114	0.29	0.049	0.012
Laetiporus sulphureus + *Laetiporus* sp.	Polyporales	99–100%	0.023	0	0	3.727	0	0	0.049	0.006
Leucogyrophana sororia	Boletales	99%	0	0	0	0	0	0	0.086	0
Leucosporidiales	Leucosporidiales	99%	0	0	0	0.017	0	0	0	0
Leucosporidiella creatinivora	Leucosporidiales	99%	0	0	0.013	0.139	0	0	0.049	0

Taxon	Order	Similarity of the reference sequence	Frequency in the fungal community (%)							
			May stumps				July–August stumps			
			< 5 cm diam.		> 5 cm diam.		< 5 cm diam.		> 5 cm diam.	
			Treated	Untreated	Treated	Untreated	Treated	Untreated	Treated	Untreated
Leucosporidium sp. + *L. drummii* + *L. fasciculatum*	Leucosporidiales	99–100%	0	0	0.404	0.417	0.019	0.011	0.012	0.143
Luellia recondita	Trechisporales	100%	0	0	0	0.121	0.038	0.129	0	0
Malassezia globosa + *M. restricta*	Incertae sedis	98–100%	0	0.37	0.013	0	0	0	0.012	0
Mastigobasidium intermedium	Leucosporidiales	100%	0	0	0	0.017	0	0	0	0
Microbotryomycetes sp.		100%	0.208	0	0.3	0.485	0.606	0.183	0.875	18.681
Microsporomyces pini	Incertae sedis	100%	0.046	0	0.104	0.104	0	0.021	0.012	0.062
Microstroma album	Microstromatales	100%	0	0.329	0.013	0.087	0.019	0.021	0.074	0.031
Mrakia frigida	Cystofilobasidiales	100%	0	0	0	0.017	0.076	0	0.037	0
Mycena cinerella + *M. galericulata* + *M. megaspora* + *M. sanguinolenta* + *M. zephirus*	Agaricales	99–100%	0.069	6.086	0.013	0.155	0	13.866	0	0.112
Naganishia cerealis	Filobasidiales	98%	0.046	0	0	0	0	0	0.062	0
Oberwinklerozyma yarrowii	Incertae sedis	99%	0	0	0	0.017	0	0.011	0.025	0.006
Papiliotrema perniciosa	Tremellales	99%	0	0	0	0.121	0	0.398	0.025	0.373
Peniophora erikssonii + *P. pini*	Russulales	99–100%	0	0	0.013	0.017	0.019	0	0.074	0
Phaeotremella skinneri	Tremellales	99%	0	0	0	0.017	0.019	0	0.049	0.056
Phanerochaete velutina	Polyporales	99%	0.254	0	0	0	0.019	0	0	0
Polyporales	Polyporales	100%	1.018	0	0.052	0	0.019	0.021	0	0.019
Postia rennyi	Polyporales	100%	0	0	0	0	0	0	0	1.344
Pseudomicrostroma phylloplanum	Microstromatales	99%	0	0	0	0	0	0.043	0.012	0.106
Pseudotremella moriformis	Tremellales	99%	0	0	0	0	0	0.064	0	0
Resinicium bicolor	Hymenochaetales	100%	0.162	0	0	0	0.019	0	0	1.238
Rhodocybe sp.	Agaricales	100%	0.093	0	0	0	0.038	0.021	0.012	0
Rhodotorula glutinis + *R. nothofagi* + *Rhodotorula* sp.	Sporidiobolales	99–100%	0.346	0	0.234	0.329	0.303	0.042	0.641	0.149
Russulaceae	Russulales	100%	0	0	0.013	0	0	0	0	0
Russulales	Russulales	100%	0	0.041	0	0.017	0	0.011	0.271	0.044
Schizophyllum sp.	Agaricales	100%	0	0	0.235	0.017	3.256	0	0.321	0
Sebacinales	Sebacinales	100%	0.254	0	0.56	0	10.356	0.215	0.049	0
Septobasidium broussonetiae + *S. pallidum*	Septobasidiales	99–100%	0.116	0	0	0.138	0.152	0.075	0.259	0.018
Sistotrema brinkmannii	Cantharellales	100%	0.046	0	0.013	0	0	0.043	11.332	5.064
Slooffia pilatii + *S. tsugae*	Incertae sedis	99–100%	0.069	0	0.026	0.035	0	0	0	0.044
Sporidiobolales	Sporidiobolales	99%	0.162	0	0.091	0.156	0.625	0.236	0.358	0.012
Sporobolomyces sp. + *S. symmetricus*	Sporidiobolales	99–100%	0.023	0	0	0	0.246	0	0.086	0
Stereum hirsutum + *S. rugosum*	Russulales	99–100%	0.532	0	0	0.034	0	0	0.123	0.087
Symmetrospora foliicola + *S. gracilis* + *Symmetrospora* sp.	Incertae sedis	99–100%	0.046	0	0	0	0.284	0.14	0.061	0.012

**Taxon**	**Order**	Similarity of the reference sequence	Frequency in the fungal community (%)							
			May stumps				July–August stumps			
			< 5 cm diam.		> 5 cm diam.		< 5 cm diam.		> 5 cm diam.	
			Treated	Untreated	Treated	Untreated	Treated	Untreated	Treated	Untreated
Tausonia pullulans	Cystofilobasidiales	99%	0	0	0	0.624	0	0.021	0.025	0
Thelephorales	Thelephorales	100%	0	0	0.026	0.035	0	0	0.037	0
Tremella sp. + *T. globispora* + *T. indecorata*	Tremellales	98–99%	0.254	0	0	0.416	0.341	0.032	0.062	0.012
Tremellales	Tremellales	99%	0.116	0	0.078	0.659	0.133	0.623	0.308	0.249
Tremellomycetes sp.	Tremellales	99%	0.278	0	0.013	1.179	0.114	0.473	0.308	0.336
Trichosporon otae + *Trichosporon* sp.	Tremellales	99–100%	0.138	3.248	0	0	0.019	0.021	0.024	0.074
Tulasnella sp.	Cantharellales	99%	0.023	0.329	0	0	0	0	0	0
Vishniacozyma carnescens + *V. globispora* + *V. victoriae.*	Tremellales	99%	0.023	0.082	0	0.208	0.019	0.247	0.037	0.049
Vonarxula javanica	Incertae sedis	99%	0	0	0.052	0.087	0.019	0	0.049	0
Xylodon raduloides	Hymenochaetales	97%	0	0	0	0	0	0	0.086	0
Yunzhangia auriculariae	Incertae sedis	100%	0	0	0	0.017	0	0	0	0.019
**Other taxa**			3.96	0.58	13.38	2.53	4.77	2.57	3.61	2.65
**Non-cultured fungi**			3.65	12.75	21.0	5.81	21.75	3.16	4.86	2.45
**Organisms without reference sequences in NCBI**			3.01	19.2	5.49	12.98	4.07	1.65	2.96	1.24
Margalef's diversity index — D_{Mg}			33.92	15.26	26.94	34.98	31.05	31.29	35.32	31.49
Shannon's diversity index — H'			2.59	2.47	2.55	3.55	3.39	3.34	3.38	3.31
Simpson's diversity index — D			0.45	0.51	0.46	0.62	0.60	0.59	0.58	0.57
Shannon's evenness index — E			0.28	0.14	0.18	0.07	0.07	0.07	0.08	0.07
Berger–Parker's dominance index — d			0.52	0.25	0.35	0.16	0.21	0.17	0.22	0.18

Percentage of frequency

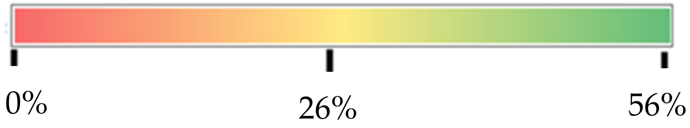

Supplement: Supplementary file 1 [file plants-09-01126-s001.pdf]
